# BLOC-1 deficiency causes alterations in amino acid profile and in phospholipid and adenosine metabolism in the postnatal mouse hippocampus

**DOI:** 10.1038/s41598-017-05465-z

**Published:** 2017-07-12

**Authors:** S. M. van Liempd, D. Cabrera, F. Y. Lee, E. González, E. C. Dell’Angelica, C. A. Ghiani, J. M. Falcon-Perez

**Affiliations:** 1 Metabolomics Platform. CIC bioGUNE, CIBER, Derio, 48260 Spain; 2 Departments of Pathology & Laboratory Medicine and Psychiatry, David Geffen School of Medicine, University of California, Los Angeles, CA 90095 USA; 3 Department of Human Genetics, David Geffen School of Medicine, University of California, Los Angeles, CA 90095 USA; 4 IKERBASQUE Research Foundation, Bilbao, Spain

## Abstract

Biogenesis of lysosome-related organelles complex-1 (BLOC-1) is a protein complex involved in the formation of endosomal tubular structures that mediates the sorting of protein cargoes to specialised compartments. In this study, we present insights into the metabolic consequences caused by BLOC-1 deficiency in pallid mice, which carry a null mutation in the *Bloc1s6* gene encoding an essential component of this complex. The metabolome of the hippocampus of pallid mice was analysed using an untargeted, liquid chromatography-coupled mass spectrometric approach. After data pre-treatment, statistical analysis and pathway enrichment, we have identified 28 metabolites that showed statistically significant changes between pallid and wild-type control. These metabolites included amino acids, nucleobase-containing compounds and lysophospholipids. Interestingly, pallid mice displayed increased hippocampal levels of the neurotransmitters glutamate and N-acetyl-aspartyl-glutamic acid (NAAG) and their precursor glutamine. Expression of the sodium-coupled neutral amino acid transporter 1 (SNAT1), which transports glutamine into neurons, was also upregulated. Conversely, levels of the neurotransmitter precursors phenylalanine and tryptophan were decreased. Interestingly, many of these changes could be mapped to overlapping metabolic pathways. The observed metabolic alterations are likely to affect neurotransmission and neuronal homeostasis and in turn could mediate the memory and behavioural impairments observed in BLOC-1-deficient mice.

## Introduction

The pallid (*pa*) mouse, originally called pinked-eyed 2, was isolated based on abnormal coat and eye colour^1^. The defective gene was identified by positional cloning and found to encode a small, coiled-coil-forming protein, named pallidin (also known as biogenesis of lysosome-related organelles complex 1 subunit 6, BLOC1S6)^2^. Besides the pigmentation defect, the pallid mouse was found to display platelet storage pool deficiency and emphysematous lung lesions. This led to the proposal that it would serve as a model for Hermansky-Pudlak syndrome (HPS)^3^. In fact, at least two HPS patients were found to carry recessive mutations in the human pallidin-encoding gene, *BLOC1S6* (formerly known as *PLDN*), thereby defining HPS type 9 (HPS-9)^4^.

Pallidin is part of the biogenesis of lysosome-related organelles complex 1 (BLOC-1), which also includes the proteins dysbindin, muted, cappuccino, snapin, BLOS1, BLOS2 and BLOS3^5, 6^. Electron microscopy revealed that the subunits of BLOC-1 are arranged in a stable elongated structure of eight globular cores^7^. There is strong evidence that all subunits are required to form a stable complex and lack of one subunit destabilizes the complex and causes a drop in levels of the other subunits, as observed in null mouse models for dysbindin (*sandy*, *sdy*), BLOS3 (*reduced pigmentation*, *rp*), muted (*mu*) and pallidin (*pa*)^6, 8, 9^.

BLOC-1 is involved in the formation of endosomal tubular structures that mediate the sorting of proteins within the endosomal-lysosomal system^10–12^. In particular, the complex controls the biogenesis of lysosome-related organelles (LROs), which comprise a group of cell-type-specific, subcellular compartments that share some features with endosomes and lysosomes and are involved in various physiological processes including pigmentation, haemostasis, lung plasticity and immunity^13^. Accordingly, mutations in the BLOC-1 complex are associated with oculocutaneous albinism, increased bleeding tendency and progressive lung disease^14^.

Besides systemic functions, BLOC-1 was also found to be relevant in a neurological context. Following reports of a possible association between common variants in the human dysbindin-encoding gene and schizophrenia^15–18^, a number of studies have found behavioural and electrophysiological abnormalities in the sandy mice (reviewed in refs 5 and 6), with a subset of these behavioural abnormalities recently reported for pallid mice as well^19^. In addition, pallid mice showed enhanced physiological and behavioural responses to morphine administration compared to the wild-type (WT) C57BL/6J strain. These effects revealed themselves by higher reduction in core temperature and increased stereotyped running^20^. Interestingly, expression levels of *BLOC1S6* and genes encoding many primary and secondary BLOC-1 binding partners such as SNAP25, Munc18-1, Synapsins and Synaptotagmin 1 were significantly lower in post-mortem nucleus accumbens samples from heroin addicts compared to matched controls^21^.

This is the first study of the effect of a defective component of the endo-lysosomal sorting machinery on the postnatal hippocampal metabolome. We evaluated the metabolic consequences of the lack of functional BLOC-1 by a comprehensive, untargeted metabolomics study of hippocampi from pallid mice. We used ultra-performance liquid chromatography-coupled Time-of-Flight mass spectroscopy (UPLC-ToF-MS or just LC-MS) in order to separate, detect and identify metabolites. After thorough statistical analysis, we found significant differences in the levels of 28 metabolites belonging to three metabolic families, namely amino acids, nucleobase-containing compounds and lysophospholipids and their derivatives. These insights have allowed us to identify affected metabolic pathways. Mechanisms through which most of these changes could be metabolically connected are discussed.

## Results and Discussion

### Mining for metabolic markers

**Figure 1:**
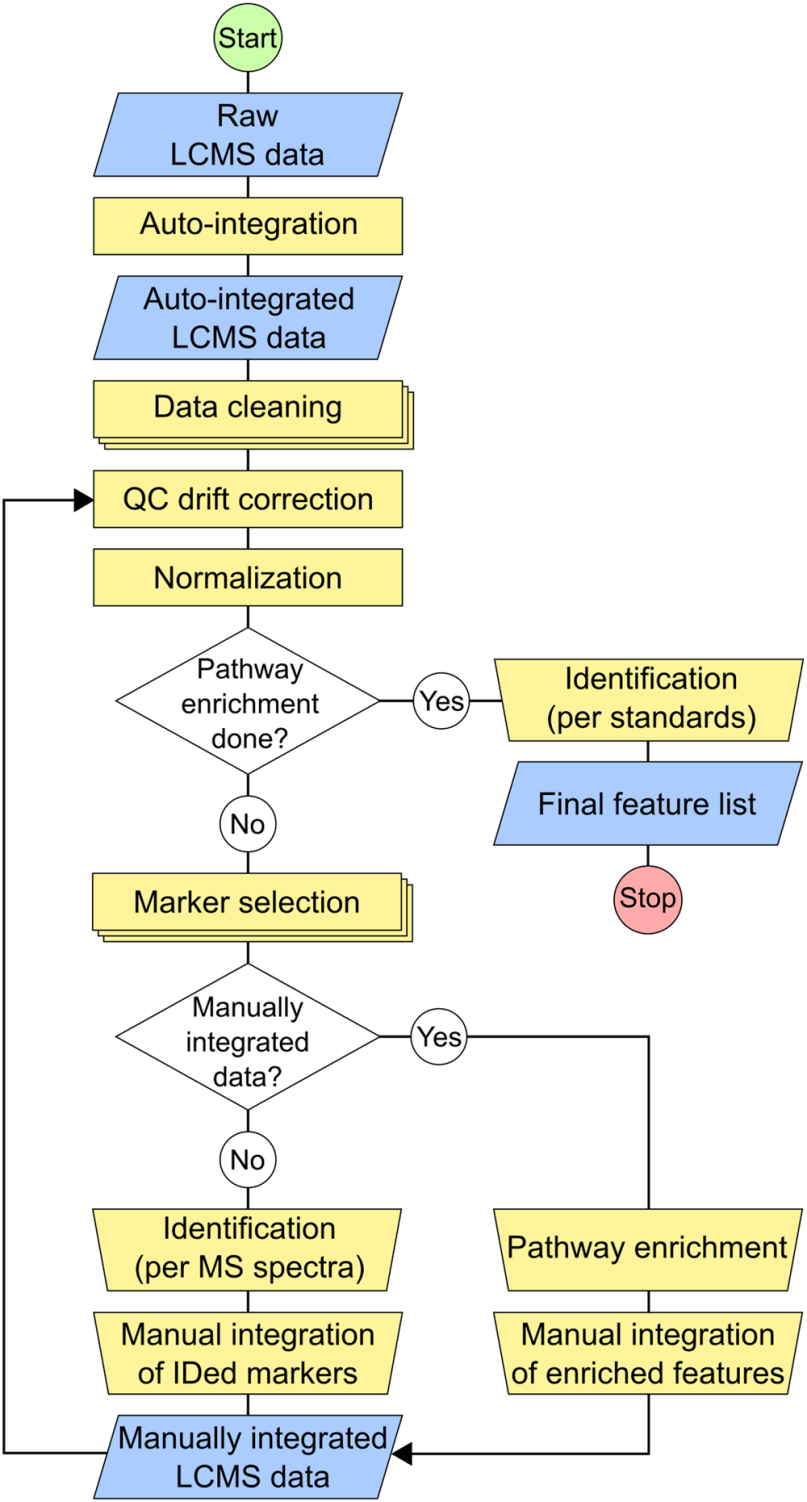
Flow chart of the data processing pipeline. Blue boxes, data types; yellow rectangles, automatic (multistage) processes; yellow trapezoids, manual processes; diamonds, decision points. A detailed description of the data processing methods is provided in Supplement 3.

Hippocampal tissues from postnatal day (P)1 and P2 WT and pallid mice were extracted in two steps obtaining an aqueous and organic extraction phase. Subsequently these phases were analysed using an untargeted LC-MS method that ran in both positive and negative ionization modes. With this approach, four raw LC-MS data-sets from the same tissue samples were obtained (*ie*. Pos/Aq, Neg/Aq, Pos/Org and Neg/Org). To identify which metabolites were affected by the pallid genotype the data were analysed following to the flow chart given in Fig. 1 (a detailed description of the data processing pipeline and its performance is given in the supplemental section).

#### Automatic data-mining

**Figure 2:**
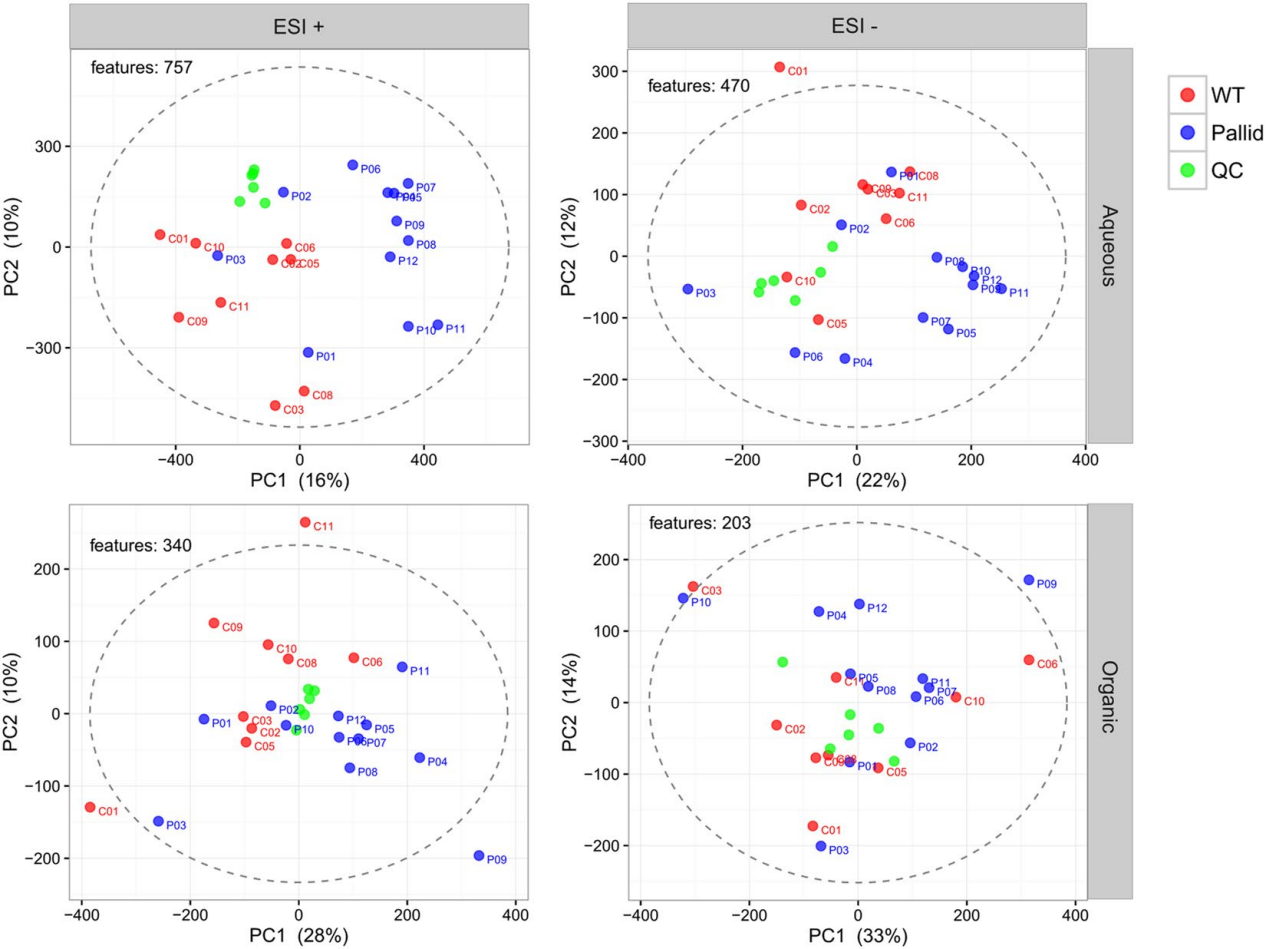
Principal component analysis (PCA) score plots representing the four different data sets. Sample scores for wild-type (WT) and pallid mice are indicated in red and blue, respectively, while the quality control (QC) samples are indicated in green. Scores are based on cleaned, QC-corrected, median-fold-change normalized, Pareto scaled and centered data. The number of features used per PCA, and the explained variance per component (PC1, PC2) are indicated in each panel. The Hotelling 95% confidence ellipses are represented by the dashed lines. ESI, electron spray ionization. Extraction phases are referred to as “Aqueous” and “Organic” as defined under Methods.

First, raw data were subjected to automatic data analysis including automatic peak integration of extracted ion-chromatograms, data clean-up, correction for signal drift and data normalization. These cleaned up and adjusted data were then used for preliminary marker selection by null hypothesis significance testing. An overview for each data-set is given as principal component score plots (Fig. 2). Here, the separation between the Pallid and WT genotypes becomes apparent. Another important observation from these score plots is that the scores for the quality control (QC) samples are clustered together more tightly than those of the extraction samples. This indicates that the observed variation was mainly due to biological rather than technical causes, expressing the soundness of the analytical and computational methods used up to this point.

#### Manual refinement

**Figure 3:**
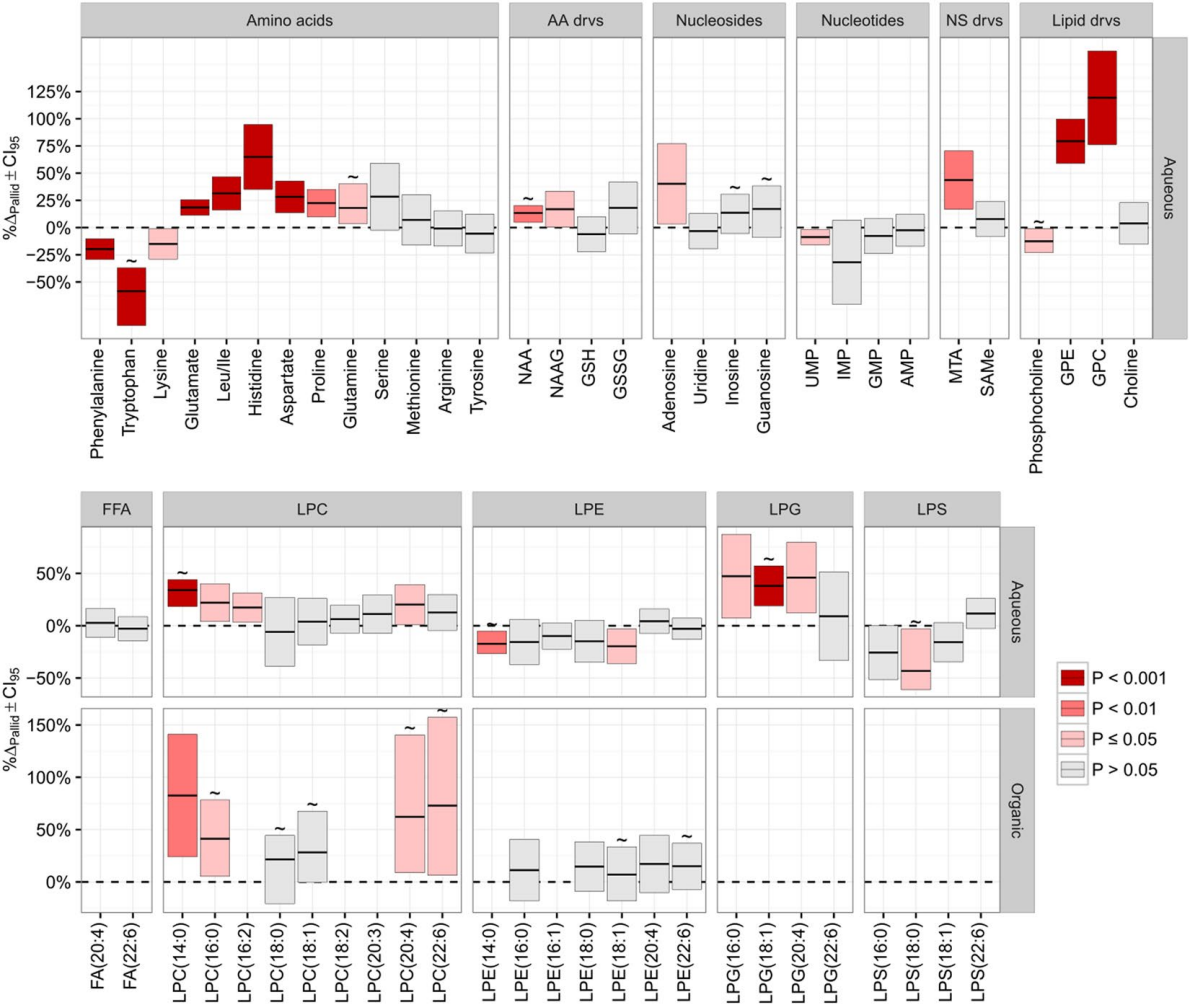
Percentage change for metabolite levels in hippocampi of pallid mice (%Δ_Pallid_ ± 95% confidence intervals) with respect to wild-type (WT) animals. The following formula was used: %Δ_Pallid_ = 100%*([Pallid] − [WT])/[WT]. Either the mean or the median (indicated by ~) values of corrected signals are used, depending on the statistical test. Confidence intervals and P values were obtained via two sample t-tests (Student’s or Welch) except for metabolites indicated with a ~ for which the statistics were calculated with the Wilcoxon rank sum test. Significance levels are color-coded using shades of red. AA, amino acids; drvs, derivatives; NS, nucleosides; FFA, free fatty acid; LPA, lysophosphatidic acid; NAA, N-acetyl-aspartate; NAAG, N-acetyl-aspartyl-glutamate; GSH, glutathione (reduced); GSSG, (oxidized); UMP, uridine monophosphate; IMP, inosine monophosphate; GMP, guanosine monophosphate; AMP, adenosine monophosphate; MTA, methylthioadenosine; SAMe, S-adenosyl-L-methionine; GPE, glycerophosphoethanolamine; GPC, glycerophosphocholine. Lipid nomenclature: H(c:b), H signifies the head group, c is the number of carbons in the acyl chain and b is the number of unsaturated bonds in the acyl chain; FA, fatty acid; LPC, lysophosphatidylcholine; LPE, lysophosphatidylethanolamine; LPG, lysophosphatidylglycerol; LPS, lysophosphatidylserine. Boxplots for the observed metabolites can be found in the Supplemental 1 and Figs S3–S5.

The second stage in the data analysis was mostly a manual laborious endeavour and consisted of identification of selected features and manual peak integration of the corresponding LC-MS signals. This resulted in a list with fourteen putatively identified marker metabolites (Table 1 and Fig. 3). This primary marker set could be roughly indexed in three groups, namely amino acids, nucleotides and lysophospholipids. Statistically significant increases (α = 0.05) were found for the amino acids aspartate, glutamate, glutamine and histidine and the neurotransmitter N-Acetylaspartylglutamic acid (NAAG), while lysine, phenylalanine and tryptophan were significantly decreased. Our findings of decreased hippocampus levels of tryptophan are in line with earlier reports of decreased levels of tryptophan and L-DOPA in the pallid brain^22^.

**Table 1:** Changes in metabolites detected in the hippocampus that differentiate the pallid from the WT genotype.

Metabolite class	Metabolite^a^	Detection method^b^	Aqueous extraction			Organic extraction		
			%*Change* ^c^	(*95*% *CIs*)	*level* ^d^	%*Change* ^c^	(*95*% *CIs*)	*level* ^d^
Amino acids	*Aspartate*	Pr	28%	(14%, 43%)	***			
	*Glutamate*	Pr	18%	(11%, 26%)	***			
	*Glutamine*	Pr	18%	(4%, 40%)	*			
	*Histidine*	Pr	65%	(35%, 95%)	***			
	(*Iso*)*leucine*	PWE	31%	(16%, 47%)	***			
	*Lysine*	PWE	−15%	(−29%, −1%)	*			
	*Phenylalanine*	Pr	−20%	(−29%, −10%)	***			
	*Proline*	PWE	22%	(10%, 35%)	**			
	*Tryptophan*	Pr	−58%	(−90%, −37%)	***			
Amino acid derivatives	*NAA*	PWE	13%	(5%, 20%)	**			
	*NAAG*	Pr	17%	(0%, 33%)	*			
Nucleosides and nucleotides	*Adenosine*	Pr	40%	(3%, 77%)	*			
	*MTA*	Pr	44%	(17%, 70%)	**			
	*UMP*	PWE	−9%	(−16%, −2%)	*			
phospholipid derivatives	*GPC*	Pr	119%	(76%, 162%)	***			
	*GPE*	Pr	79%	(59%, 100%)	***			
	*Phosphocholine*	PWE	−13%	(−23%, −1%)	*			
	*LPC*(*14*:*0*)	Pr	34%	(18%, 44%)	***	83%	(24%, 141%)	**
	*LPC*(*16*:*0*)	Pr	22%	(4%, 40%)	*	41%	(5%, 78%)	*
	*LPC*(*16*:*2*)	PWE	17%	(3%, 31%)	*			
	*LPC*(*20*:*4*)	Pr	20%	(1%, 39%)	*	62%	(9%, 141%)	*
	*LPC*(*22*:*6*)	PWE				73%	(6%, 157%)	*
	*LPE*(*14*:*0*)	PWE	−17%	(−27%, −5%)	**			
	*LPE*(*18*:*1*)	PWE	−20%	(−36%, −3%)	*			
	*LPG*(*16*:*0*)	PWE	47%	(7%, 87%)	*			
	*LPG*(*18*:*1*)	PWE	38%	(19%, 57%)	***			
	*LPG*(*20*:*4*)	PWE	46%	(12%, 80%)	*			
	*LPS*(*18*:*0*)	PWE	−43%	(−61%, −3%)	*			

^a^NAA: N-acetylaspartate, NAAG: N-Acetylaspartylglutamic acid, MTA: methylthioadenosine, UMP: uridine monophosphate, GPE: glycerophosphoethanolamine, GPC: glycerophosphocholine, LPA: lysophosphatidic acid, LPC: lysophosphatidylcholine, LPE, lysophosphatidylethanolamine, LPG: lysophosphatidylglycerol, LPS: lysophosphatidylserine. For the lysophospholipids the numbers between brackets indicate the amount of carbons and the amount of unsaturated bonds, respectively, in the fatty acid moiety.

^b^Pr: present in the auto-integrated primary data set. PWE: detected via pathway enrichment.

^c^%Change = 100 × Δ/〈X〉_WT_, where Δ is the difference (pallid – WT) in means (t-test) or location parameters (Wilcoxon rank sum test). 〈X〉_WT_ is either the mean in the WT (t-test) or median (Wilcoxon rank sum test).

^d^Level: ***P < 0.001; **P < 0.01; *P ≤ 0.05.

Among the nucleosides, adenosine and methylthioadenosine (MTA) were upregulated in the pallid hippocampus. Finally, glycerophosphocholine (GPC), glycerophosphoethanolamine (GPE) and the lysophosphatidylcholines (LPCs) LPC(14:0), LPC(16:0), LPC(20:4) were upregulated as well.

#### Pathway enrichment

In the third and last stage, the metabolic pathways in which these markers were present were identified. By searching the LC-MS data for nearest neighbours of the markers in the identified pathways, 14 additional metabolite markers and 29 unaltered metabolites were added to the final feature list. Thus, a total of 28 metabolite markers between extraction phases and polarities were found (Table 1 and Fig. 3).

For both the organic and aqueous extraction phases, LPCs and lysophosphatidylethanolamines (LPEs) were detected after pathway enrichment. However, only LPCs exhibited upregulation in both phases, while LPEs were upregulated only in the aqueous phase. Lysophosphatidylglycerol (LPG) and lysophosphatidylserine (LPS) were only detected (and found to be differentially expressed) in the aqueous extractions. Besides lysophospholipids, changes in the levels of proline, N-acetylaspartate (NAA), uridine monophosphate (UMP) and phosphocholine were also found via pathway enrichment.

Finally, most metabolite identities were confirmed using chemical standards. Boxplots for all analysed metabolites based on adjusted signal intensities are included in the supplemental section (Supplemental 1 and Figs S3–S5), together with detailed information about spectrometric properties and identifications of all included metabolites (markers and non-markers). Metrics for each step of the data mining process are also included in the supplemental section (Table S1).

### Organic versus aqueous extractions

The fact that most metabolic changes were observed in the aqueous extractions may simply reflect the fact that most metabolites (differentially expressed or not) were detected in this phase. The aqueous extraction method was thought to extract the soluble component from the cytosol and the lumen of ruptured organelles. Thus, the pellet, which was left after aqueous extraction, was thought to contain precipitated proteins and membrane fragments. Sequential extraction of this pellet with chloroform should then result in further dissolving of metabolites included in the precipitated structures, including LPEs and LPCs. The results for LPCs in the organic extraction are very similar to those in the aqueous extraction method with respect to change but not to peak intensities (Figs S4 *vs*. S5). This could indicate different amounts of LPCs in cytosol and precipitate, with higher amounts in the cytosolic fraction. In contrast, the levels of LPE(18:1) in the organic extraction did not differ significantly between genotypes. LPE(14:0), which showed the most pronounced changes in the aqueous extraction, was not detected in the chloroform extraction. However, although the P values were well above 0.05 for all observed LPEs in the organic extractions, on average the levels seemed higher in pallid. This might indicate differences in lipid metabolism between soluble and membrane fractions.

### Possible mechanisms underlying the altered pallid metabolome

The 28 differentially expressed metabolites found in pallid hippocampal tissue could be classified in three distinct metabolic classes, namely: amino acids, nucleobase-containing compounds and lysophospholipids and derivates. These findings suggest that BLOC-1 deficiency has a pleiotropic impact on metabolism. In the following sections, we map the observed metabolites to canonical metabolic pathways in the brain to explain these changes from a metabolic point of view. Remarkably, an overlap between the three metabolic classes can be revealed.

#### Amino acids

**Figure 4:**
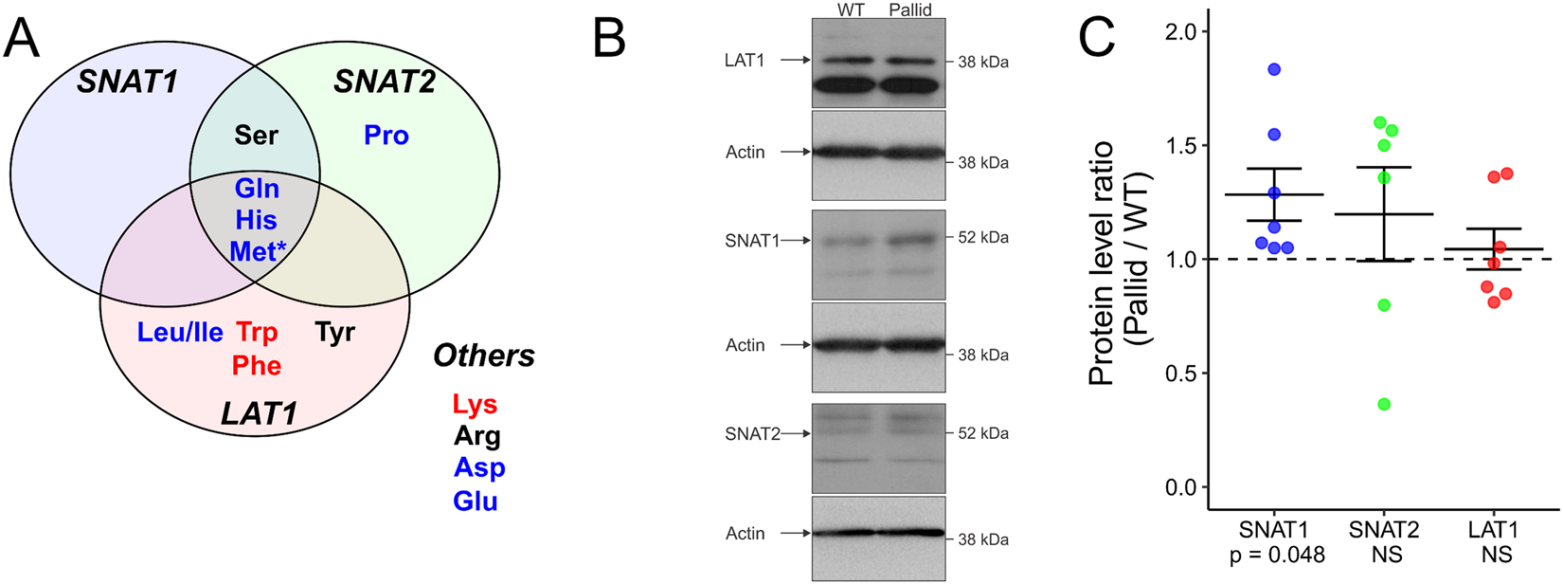
Changes in amino acid transporters and substrates in the hippocampus of pallid mice. (**A**) Substrate specificity of the detected amino acids for System A (SNAT1 and SNAT2), System L (LAT1) or other (Others) amino acid transporter systems. Amino acid names in blue and red are upregulated and downregulated in pallid hippocampi, respectively. *While levels of methionine (Met) are not significantly changed, its product metabolite methylthioadenosine (MTA) is upregulated in pallid mice. (**B**) Representative immunoblots of whole-tissue extracts prepared from hippocampi of P1 wild-type (WT) or pallid mice, probed with antibodies to LAT1, SNAT1 or SNAT2, and subsequently re-probed with an antibody to β-actin. All sets of immunoblots are shown in Supplemental 1 and Fig. S6. The pairs of WT and pallid samples used in panel B for LAT1, SNAT1 and SNAT2 correspond to sets 2, 1 and 3 in Supplemental 1 and Fig. S6, respectively. (**C**) Values derived from densitometric analysis were background-corrected, normalized to those obtained for β-actin, and are represented as a Pallid/WT ratio for each pair of hippocampi extracts that was processed in parallel. Indicated are the means ± SEM (standard error of the mean) of 6 to 7 pairs of WT and pallid animals. P values were derived from 2-tail one-sample t-tests of each data group against the theoretical value of 1 (dashed line). When P values were higher than 0.05 differences were deemed not significant (NS). Notice a small but statistically significant upregulation of SNAT1 levels in pallid hippocampi compared to wild-type (WT) control. No significant changes were found in LAT1 expression levels, while SNAT2 levels display a modest non-significant increase compared to WT but also a high variability.

Amino acid levels in brain cerebrospinal and interstitial fluids differ greatly from those found in the periphery. Brain amino acid levels are much lower than those in plasma, except for glutamine, which is only slightly reduced in the brain^23, 24^. Brain homeostasis of amino acids is secured by a range of transporter systems present on the blood-brain barrier (BBB). Based on the changes in the hippocampal amino acid profile of pallid mice, the System L and System A transporter systems deserved our attention. Both systems are present in the BBB and share substrates that were found at altered levels in pallid hippocampus (Fig. 4A).

The large neutral amino acid transporter 1 (System L, SLC3A2/SLC7A5, 4F2hc/LAT1, herein referred to as LAT1) transports glutamine, histidine (iso-)leucine methionine and other large neutral amino acids and is the sole transporter of aromatic amino acids tryptophan and phenylalanine across the BBB^25^. Moreover LAT1, present at both sides of the BBB^26^, is thought to be primarily responsible for glutamine brain homeostasis^23^. LAT1 is an obligatory anti-porter, which means that it must exchange one intracellular substrate for an extracellular one. Besides expression in the BBB, it is also found in neurons and astrocytes.

The sodium-dependent neutral amino acid transporters 1 and 2 (System A, ATA1/2, SAT1/2, SLC38A1/2, herein referred to as SNAT1 and SNAT2) transport glutamine, histidine, proline and methionine, amongst others. Unlike LAT1, SNATs exclusively transport extracellular substrates together with Na^+^ to the cytosol in exchange for intracellular K^+^. While SNAT1 is brain specific and primarily expressed by, but not confined to, neurons^27^, SNAT2 is found in the BBB endothelium, neurons and astrocytes and is ubiquitously expressed throughout the rest of the body^28–30^.

The relative protein expression levels of L and A type transporters in hippocampi from P1 pallid and WT mice were determined by Western blotting (Fig. 4B and C). Despite our attempts to minimize the variability between samples, for instance by using pairwise analysis of pallid and WT samples that had been processed side-by-side throughout the entire experiment (starting with tissue homogenization), a high variability of relative protein expression levels was noted, particularly for SNAT2 (Fig. 4C). Consequently, no statistically significant differences were observed for SNAT2 and LAT1. On the other hand, the protein levels of SNAT1 displayed a small (~28%) but statistically significant (α = 0.05) increase in P1 pallid hippocampi as compared to WT mice (Fig. 4C). Not necessarily such a modest increase in steady-state levels of SNAT1 protein may underlie mechanistically the alterations in amino acids seen in the pallid hippocampi. Interestingly, the expression of genes encoding pallidin and SNAT1 could be functionally linked via the epigenetic regulator methyl-CpG binding protein 2 (MeCP2), since MeCP2 deficiency has been reported to lead to a reduction in pallidin levels in the hippocampus^31^ and higher SNAT1 densities in astrocytes^32^ and microglia (but not in glutamatergic neurons)^33^.

Glutamine is one of the substrates shared between Systems L and A and is used in the production of glutamate and aspartate in glutamatergic neurons^34, 35^. These amino acids are in turn the precursors of the neuropeptides NAA and NAAG, which are also primarily produced in neurons^36^. Synthesis of glutamate, aspartate, NAA and NAAG are neuronal processes using glutamine as a precursor, thus neuronal influx of glutamine must be increased. Glutamate is subsequently transported into synaptic vesicles, and is excreted upon an action potential to facilitate excitatory neurotransmission. It is at this level that glutamate accumulation might take place. In support of this notion, abnormal glutamatergic neurotransmission has been reported in the sandy mouse, another mouse model of BLOC-1 deficiency, and has been attributed to defects in the priming and trafficking of synaptic vesicles by reduced expression of L and N-type calcium channels and consequently reduced intracellular Ca^2+^ ^37^. Since the proper assembly and function of BLOC-1 depends on the presence of all of its subunits, it is conceivable that a deficiency in synaptic vesicle transport in pallid mice could lead to the observed metabolic hippocampal phenotype. For instance, glutamate filled vesicles could accumulate because of a faulty releasing mechanism, thereby increasing glutamate and (lyso)phospholipid pools. In order to compensate for reduced glutamate signalling, neuronal glutamine influx could be increased by upregulation of SNAT1 transporters.

It must be stressed that amino acid regulation in the brain is a very complex process that involves many transporter systems with overlapping substrate preferences. In our analyses only a subset of amino acids was detected and only expression of some, although important, transporters were probed, thus, we should consider that other transport and metabolic processes are likely to be affected as well. This is exemplified by unchanged levels of tyrosine, which is a System L substrate, or by the decreased levels of lysine, which is transported by System y^+^. Further research will be necessary to clarify this point.

#### Nucleobase-containing metabolites

The nucleoside adenosine and the sulphur-containing nucleotide derivative methylthioadenosine (MTA) were found to be upregulated in hippocampal tissue from pallid mice, while the nucleotide uridine monophosphate (UMP) showed a slight but significant downregulation.

**Figure 5:**
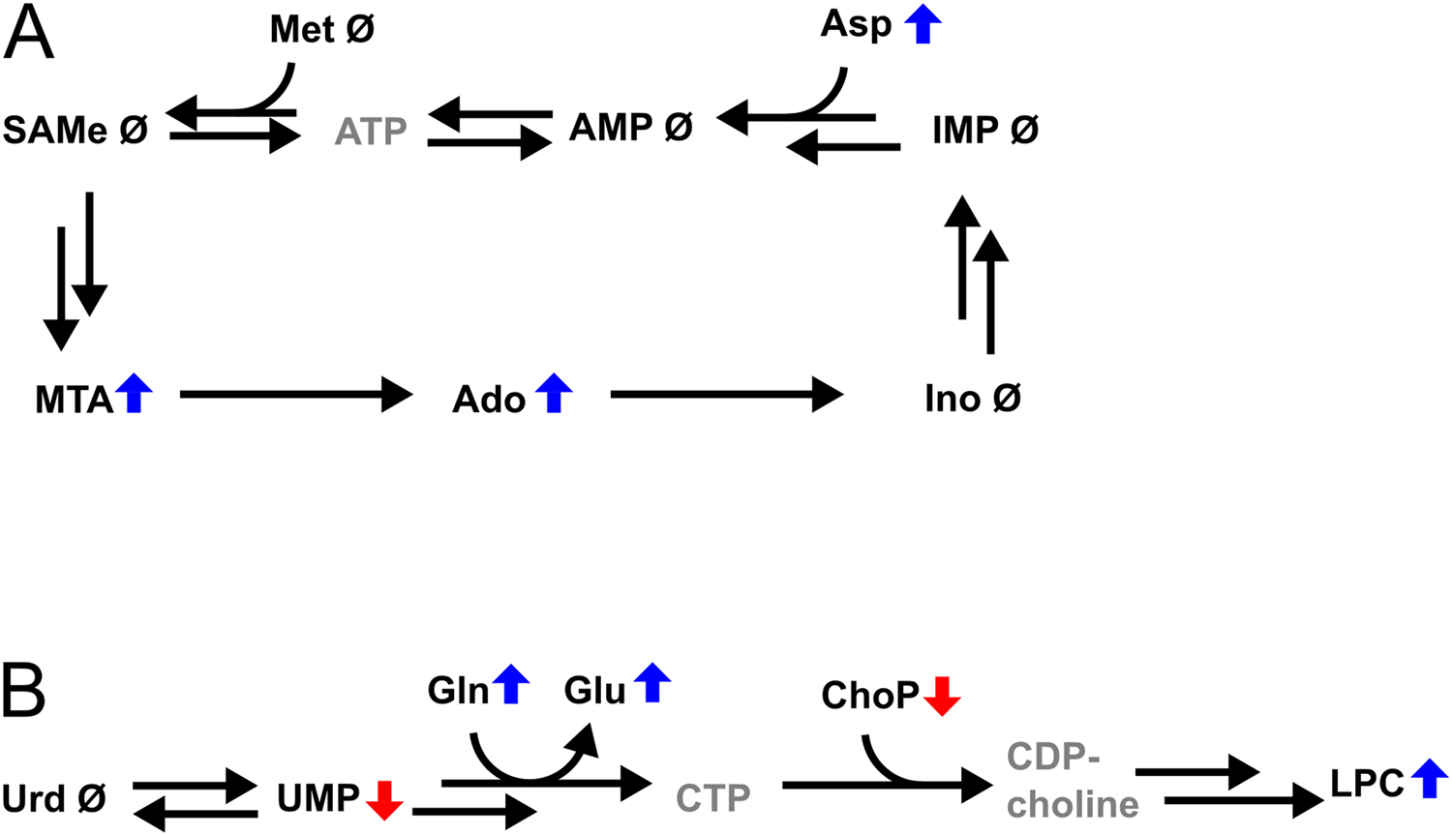
Changes in metabolism of nucleobase-containing compounds in the hippocampus of pallid mice. (**A**) Overview of changes in purine nucleosides and the adenosine salvage pathway via MTA. Ado, adenosine; Ino, inosine; AMP, adenosine monophosphate; ATP, adenosine triphosphate; Met, methionine; SAMe, S-adenosyl-L-methionine; MTA, methylthioadenosine; Ado, adenosine; Ino, inosine; IMP, inosine monophosphate. (**B**) Overview of changes in the pyrimidine nucleoside/nucleotide pathway and lysophospholipid synthesis. Urd, uridine; UMP, uridine monophosphate; CTP, cytidine triphosphate; CDP-choline, cytidine diphosphate choline; ChoP, phosphocholine; LPC, lysophosphatidylcholine; Gln, glutamine; Glu, glutamate. Blue arrows, upregulation in hippocampus of pallid mice; red arrow, downregulation; Ø, no change. Greyed-out metabolites were not observed or quantifiable.

Upregulation of adenosine could be explained by increased activity of the methionine/adenosine salvage pathway via MTA (Fig. 5A)^38^. In this pathway, ATP reacts with methionine to form S-adenosyl-L-methionine (SAMe), which can be converted to MTA. MTA can then be converted back to adenosine. Interestingly, the only shared System A/L substrate that did not show a significant change in pallid was methionine. However, its product metabolite MTA was found increased. Upregulation of this salvage pathway would require, besides increased availability of methionine, also increased production of adenosine monophosphate (AMP). AMP is produced via inosine monophosphate (IMP) with aspartate as a nitrogen donor^39^ (Fig. 5A). This step could be facilitated by an increased concentration of aspartate as is the case in pallid hippocampi. Although not significant, IMP levels seemed to be slightly reduced in pallid. The steady-state WT-baseline levels of methionine, AMP and SAMe could be maintained in pallid via this salvage pathway.

Furthermore, downregulation of UMP in pallid might be correlated with changes in phospholipid metabolism, which could be explained as follows. Glutamine is used in the conversion of uridine triphosphate (UTP) into cytidine triphosphate (CTP, Fig. 5B)^40^. Since UTP is derived from UMP, decreased levels of UMP, as found in pallid hippocampi, could reflect the increased production of CTP. CTP, in turn, can react with phosphocholine (ChoP, Fig. 5B), which was decreased in pallid, to produce CDP-choline. The latter metabolite is used in the synthesis of phosphatidylcholine (PC). Via the action of phospholipase A, PCs are metabolised to lysophosphatidylcholine. Hence, upregulated glutamine levels might facilitate the production of phospholipids via UMP and phosphocholine.

#### Lysolipids

**Figure 6:**
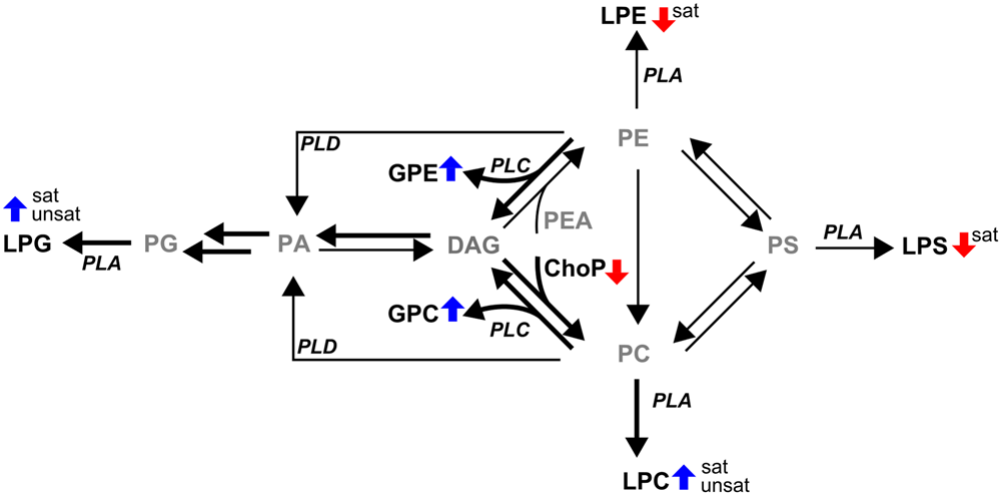
Overview of changes in lipid metabolism in the hippocampus of pallid mice and a proposed mechanism by which the altered lipid profile in pallid could be explained. The pathways indicated by thick arrows are thought to be more active in pallid based on observed changes in metabolites. (L)PS, (lyso)phosphatidylserine; (L)PE, (lyso)phosphatidylethanolamine; (L)PC, (lyso)phosphatidylcholine; (L)PG, (lyso)phosphatidylglycerol; DAG, diacylglycerol; PEA, phosphoethanolamine; GPE, glycerophosphoethanolamine; GPC, glycerophosphatidylcholine; ChoP, phosphocholine; PLA/C/D, phospholipase A/C/D. Blue arrows, upregulation in hippocampus of pallid mice; red arrow, downregulation; Ø, no change; unsat, poly-unsaturated acyl-groups; sat, saturated acyl-groups. Greyed-out metabolites were not observed or quantifiable.

Due to the high complexity of phospholipid synthesis (reviewed in ref. 41) and the fact that we could only quantify the lyso-forms rather than the diacylated species, it is challenging to explain the observed differences. However, by using canonical lipid metabolism pathways we could rationalize changes in lysophospholipids and their derivatives (Fig. 6).

Increased levels of GPC and GPE, as observed in pallid, could indicate an increased activity of phospholipase C (PLC). This enzyme cleaves head groups of PC and phosphatidylethanolamine (PE) resulting in the formation of GPC and GPE, respectively, and diacylglycerol (DAG) (Fig. 6). PLC is implicated in the response to excess production of phospholipids^42^. Therefore, elevated PLC activity could act as a compensation mechanism for the increased levels of PC, as discussed in the previous section.

Consequently, since phosphatidylserine (PS) can be derived from PE and the latter being catabolised by PLC into GPE and DAG, it is possible that decreased PE levels lead to decreased PS levels as indicated by the decrease in LPE and LPS. It should be noted that phosphoethanolamine (PEA), which is the equivalent of phosphocholine in the synthesis of PEs, was not detected in either of the genotypes. Yet, MS ionisation efficiencies of phosphoethanolamine and phosphocholine are expected to be in the same range (equivalent to GPC/GPE). Therefore, it is possible that in general, PE synthesis in hippocampus is channelled via the conversion of PS rather than via DAG and that a sink in PE causes an increased conversion and lower levels of PS.

Finally, increased LPG levels could also be explained by increased LPC activity. As mentioned, increased GPE and GPC levels indicate increased activity of PLC and production of DAG. DAG can be converted to phosphatidic acid which, after reaction with CTP (assumed to be increased in pallid), can be converted to phosphatidylglycerol. Therefore, upregulation of this route can explain the elevated levels of LPG (via PLA) in pallid mice (Fig. 6).

In conclusion, we suggest that the changes in phospholipid metabolism are due to a compensatory effect, initiated by the excess production of PC and subsequent activation of PLC.

### Neuropathological relevance of the metabolic changes in pallid mouse

The increase in glutamate levels is likely the most noticeable metabolic effect observed in the pallid genotype. Glutamate is the major excitatory neurotransmitter in the central nervous system, and hippocampal glutamatergic pathways are involved in processes underlying memory and learning. From a neuropathophysiological perspective, glutamate is of importance because altered glutamatergic transduction has been implicated in the neurochemical mechanism behind schizophrenia^43^. Interestingly, the sandy mouse, another model of BLOC-1 deficiency, shows reduced glutamate signalling. More specifically, this mouse model carries a mutation in the gene encoding dysbindin, which has been associated with schizophrenia in humans. Likewise, NAAG is another neurotransmitter found to be increased in pallid hippocampus which might be involved in schizophrenia aetiology^44^. Moreover, the decreased levels of tryptophan and phenylalanine found in the pallid hippocampus could affect neurotransmission. These amino acids are the precursors for serotonin and melatonin (via tryptophan) and dopamine (via phenylalanine), and dysfunction of the latter has been implicated in schizophrenia^43^. In the context of the observed opiate sensitivity in pallid mice^20^, changes in glutamate might also be of interest. Glutamatergic neurotransmission in the hippocampus has been shown to be necessary for opiate reward, as reviewed in Peters *et al*.^45^. Finally, accumulation of lysophospholipids could have detergent-like effect on neuronal membranes leading to neuronal damage. What is more, since they are precursors in the formation of platelet activating factor, increased levels of lysophospholipids could also trigger neuro-inflammation^46^.

## Conclusion

By applying an unbiased and comprehensive LC-MS-based metabolic profiling approach to hippocampi of postnatal pallid mice, we found statistically significant changes in the levels of amino acids, nucleosides and lipids suggesting that BLOC-1 deficiency has pleiotropic effects on postnatal hippocampal metabolism. Future studies will be required to determine whether these changes extend to other parts of the brain, *e*.*g*. cortex, and/or continue throughout later postnatal development into adulthood.

The most important observations were the increased levels of glutamate and NAAG and the decreased levels of tryptophan and phenylalanine, since these are either neurotransmitters or precursors thereof. Changes found in amino acids might be associated with alterations in the homeostasis of the sodium-coupled neutral amino acid transporter SNAT1, which transport glutamine among other substrates. These metabolic alterations could, in turn, affect neurotransmission and neuronal homeostasis, triggering the cognitive and behavioural impairments observed in BLOC-1-deficient mouse models. The association with schizophrenia, opiate addiction and neuro-inflammatory processes in combination with the detailed metabolomic map presented in this study, could make the pallid mouse an interesting tool to unravel the complex mechanisms underlining these neurological afflictions.

## Methods

Metabolomics studies combine a vast array of materials, analytical and computational methods in order to find differentiating metabolites. For sake of readability, only a minimal description of the biological materials, analytical methods and data processing are included here. A complete overview of the materials and methods and the data processing steps, including metrics for each step, are presented in the supplemental section.

### Hippocampal tissue extraction

BLOC-1-deficient pallid mutant mice (B6.Cg-*Bloc1s6* ^*pa*^/J) and the WT control strain (C57BL/6J) were bred and maintained at the University of California, Los Angeles (UCLA). All experimental procedures involving vertebrate animals, namely euthanasia and dissection of hippocampi, were carried out in accordance to the guidelines and policies of the UCLA Chancellor’s Animal Research Committee, following review and approval by the UCLA Office of Animal Research Oversight. Hippocampi were dissected from pallid and WT mice at P1 or P2 as described previously^9^ and rapidly flash frozen. The frozen tissue samples were then shipped on dry ice to the metabolomics platform of CIC bioGUNE in Derio, Spain, where they were stored at −80 °C until further use. In total, 9 WT and 12 pallid samples were used in this study.

### Sample preparation and LC-MS analysis

In a first extraction step, whole hippocampus preparations were bead-homogenized in 500 µL of ice cold 50% (v/v) MeOH/water. After solvent evaporation the resulting pellets were resuspended in 100 µL 50% (v/v) MeOH/water. These samples are further referred to as the aqueous extractions. The pellets obtained after homogenization and removal of the supernatant were further extracted in 3:1 (v/v) chloroform/MeOH. The resulting suspensions were evaporated to dryness and resuspended in 100 µL MeOH. These samples are thus referred to as the organic extractions.

Samples were analysed on a UPLC system (Acquity, Waters Inc., Manchester, UK) coupled to a time-of-flight mass spectrometer (ToF-MS, SYNAPT G2, Waters Inc). The MS was operated in both positive and negative electrospray ionization (ESI+/−) modes. Since each extraction phase was run in both polarities, four data-sets were obtained, *i*.*e*. ESI+/aqueous extraction (Pos/Aq), ESI+/organic extraction (Pos/Org), ESI−/aqueous extraction (Neg/Aq) and ESI−/organic extraction (Neg/Org).

All samples were separated on a BEH C18 UPLC column (Waters Inc.) in a linear gradient of water acetonitrile and formic acid. The flow rate was 140 µL/min and the injection volume was 5 µL. Injection sequences consisted of test mixes, extraction controls, initialization runs, randomized sample sequences and QCs. Furthermore 6 quality control samples per extraction phase and polarity were recorded.

### Data processing

A graphical overview of the data processing steps is given in Fig. 1. In short, automatically integrated LC-MS data (MarkerLynx, Waters Inc) was cleaned from non-endogenous, background noise signals. The remaining signals were corrected for drift if detected in QC samples. Next, the signals were normalized with the median fold change method^47, 48^. Adjusted data were subjected to null hypothesis significance testing by either two-sample t-test or Wilcoxon signed rank test, depending on the outcome of the Shapiro-Wilk test for normality. Also taken in account was the homogeneity of variance via the Barlett test. Data adjustments and statistical analysis were done in R (R 3.2.3, R Core Team, 2015).

After this first selection, rough identifications based on exact mass to charge ratios (*m*/*z*), isotope distributions, fragmentation patterns and database hits (HMDB, METLIN) were performed. Peaks in the extracted ion-chromatograms of features with a putative identification were manually reintegrated (QuanLynx, Waters Inc.) and the new values were again subjected to data adjustments and significance testing. The metabolite markers from this second selection were used for pathway enrichment. The enriched feature list was subjected to a final identification step with chemical standards.

### Western-blot analysis

Whole tissue lysates were prepared from pairs of P1 hippocampi from pallid and WT animals (n = 6 to 7 pairs of pallid and WT) as previously reported^9^. Total protein concentration in cleared extracts was estimated using the ThermoScientific^TM^ Pierce^TM^ BCA Protein Assay Kit (Thermo Fisher Scientific, Waltham, MA). Thirty-five μg of total proteins were loaded on a 4–12% Tris-Glycine gel (Invitrogen, Carlsbad, CA) and immunoblotting was performed as previously described^9^. Equal protein loading was verified by Ponceau S solution (Sigma, St. Louis, MO) reversible staining of the membranes, and each extract was also analysed for relative protein levels of β-actin by stripping and re-probing. Membranes were incubated for 48 h at 4 °C with the primary antibodies, followed by 30 minutes at RT with the appropriate HRP-conjugated secondary antibody. Protein bands were detected by chemiluminescence using the Thermo Scientific™ Pierce™ ECL 2 Western Blotting Substrates (SNAT1), the GE Healthcare Amersham™ ECL™ Prime (LAT1 and SNAT2) or ECL™ kit (β-actin) Western Blotting detection reagents (GE Healthcare, Piscataway, NJ). Relative intensities of the protein bands were quantified by scanning densitometry using the NIH Image Software (Image J, http://rsb.info.nih.gov/ij/) and each value background corrected. For the comparison of relative protein levels, each background-corrected value was normalized to the relative levels of β-actin of the sample. Data are shown as pallid/WT ratio of 6 to 7 pairs of pallid and WT extracts, which were prepared and analysed in parallel. Statistical analysis was performed using GraphPad Prism 7.0b (GraphPad Software; San Diego, CA) by one sample t-test. A P value less than 0.05 was considered significant.

## Electronic supplementary material

Supplemental information

Dataset 1
