# Supplementary material for: BLOC-1 deficiency causes alterations in amino acid profile and in phospholipid and adenosine metabolism in the postnatal mouse hippocampus
Source: Sci Rep. 2017 Jul 12;7:5231. doi: 10.1038/s41598-017-05465-z (PMC5507893; doi:10.1038/s41598-017-05465-z)
Supplement: Supplementary file 1 — Supplemental information [file 41598_2017_5465_MOESM1_ESM.pdf]

SUPPLEMENTAL INFORMATION

BLOC-1 deficiency causes alterations in amino acid profile and in phospholipid and adenosine metabolism in the postnatal mouse hippocampus.

Authors

S. M. van Liempd[1,*], D. Cabrera[1], F. Y. Lee[2], E. González[1], E.C. Dell´Angelica[3] , C.A. Ghiani[2] and J.M. Falcon-Perez[1,4,*]

Affiliations

[1] Metabolomics Platform. CIC bioGUNE, CIBER, Derio, 48260, Spain

[2] Departments of Pathology & Laboratory Medicine and Psychiatry, David Geffen School of Medicine, University of California, Los Angeles, CA 90095, USA.

[3] Department of Human Genetics, David Geffen School of Medicine, University of California, Los Angeles, CA 90095, USA

[4] IKERBASQUE Research Foundation, Bilbao, Spain

* smvanliempd@cicbiogune.es, jfalcon@cicbiogune.es

CONTAINS:

Supplemental 1: Figures S1-S6

Supplemental 2: R-script

Supplemental 3: Additional Methods and Results

Supplemental 4: Table S1

Supplemental 1 - Figures

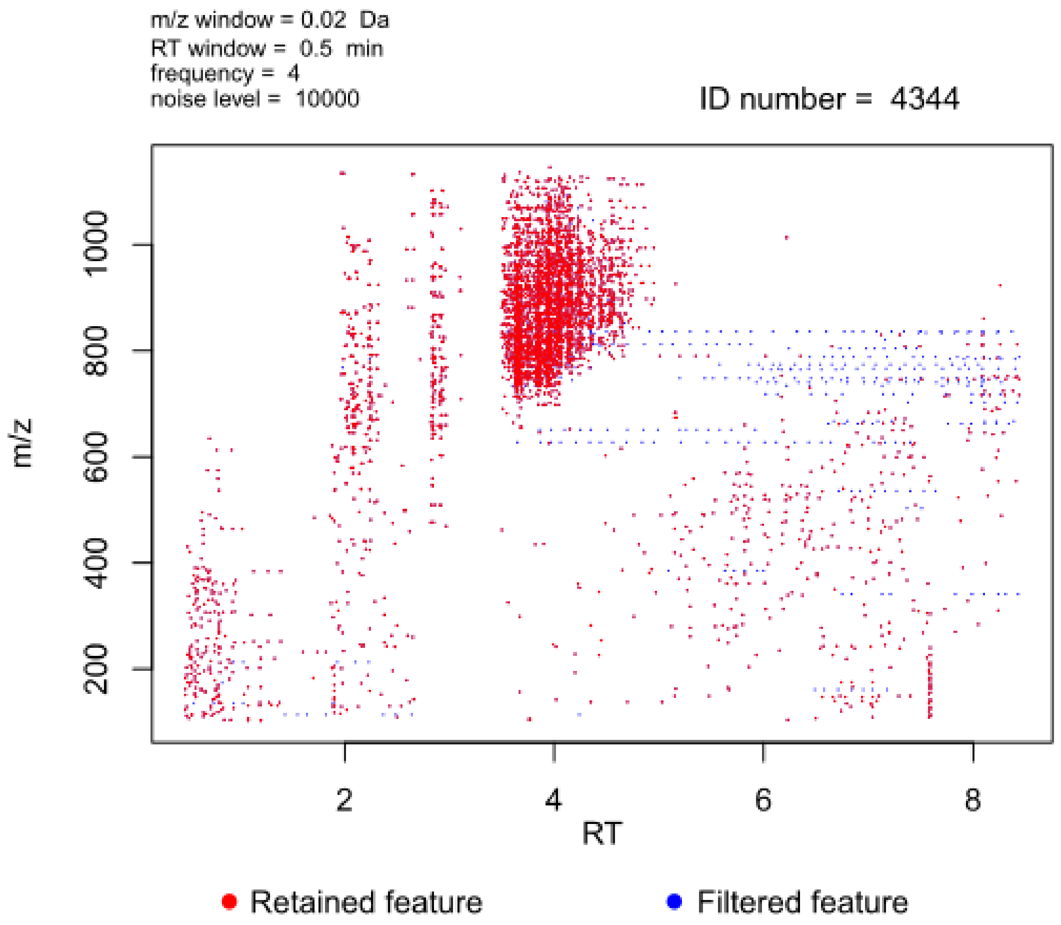

Figure S1) Performance of heuristic noise filtering. Plot showing retention times (RT in minutes) vs *m/z* values of retained (red) and excluded (blue) features for the Pos/Aq data set. All features in an *m/z* window of 20 mDa, repeated 4 times or more in a time window of 30 s with intensities under 10000 are excluded.

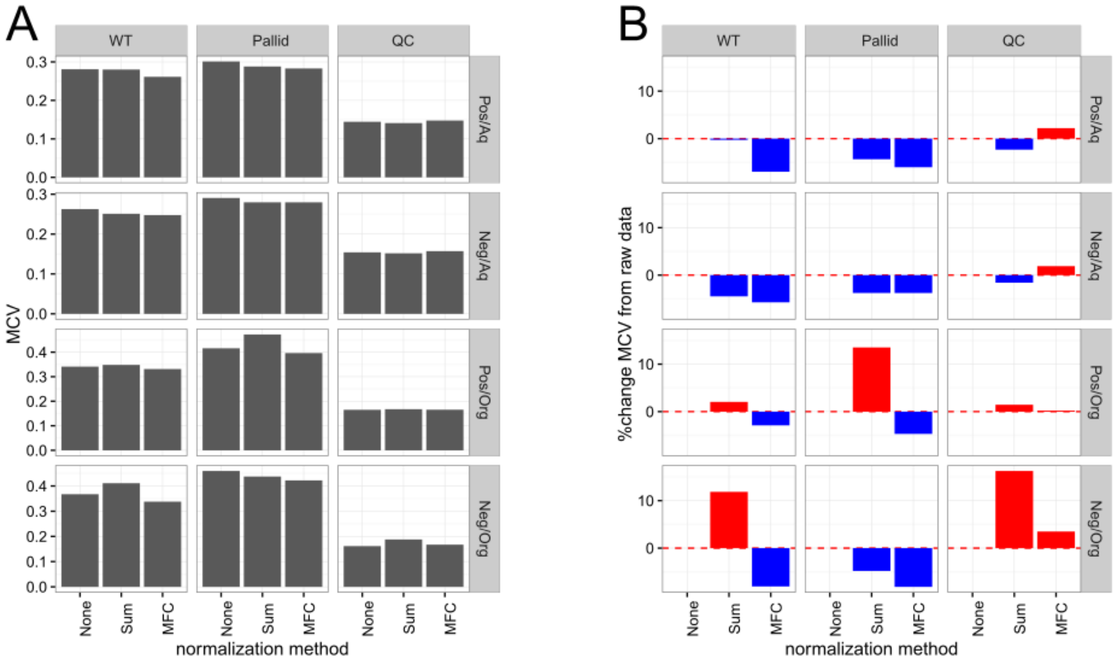

Figure S2) Comparisons of the medians of the CV (coefficient of variation) values (MCV) in the three sample groups (WT, Pallid, QC) of features that were left after data clean-up for different normalization methods (None, sum, MFC). The 'None' method is just the MCVs from the QC-corrected data, the 'Sum' method is normalizing the data on the total sum of the features per sample and the 'MFC' method normalizes the median fold change. Panel A contains the absolute MCV values for each sample group and normalization method, while panel B shows the relative differences compared the non-normalized data.

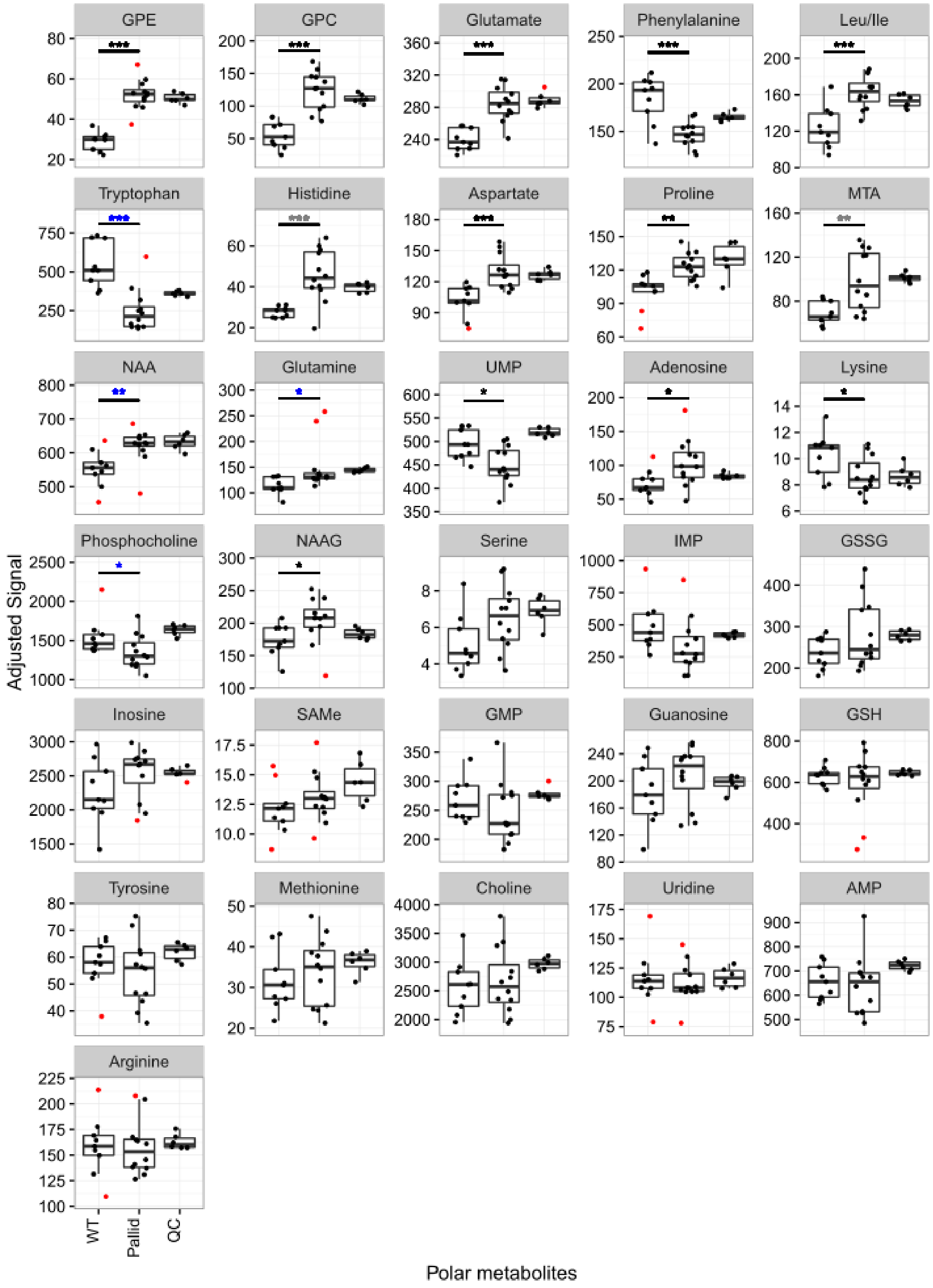

Figure S3) Boxplots for polar metabolites (amino acids and derivatives, nucleosides, and phospholipid derivatives). QC: quality control. Signals are the QC-corrected, normalized (MFC method) areas of manually integrated peaks. Significance levels: *** $p < 0.001$, ** $p < 0.01$, * $p \leq 0.05$. Black stars, Student’s Two Sample t-test; grey stars, Welch Two Sample t-test; blue stars, Wilcoxon rank sum test.

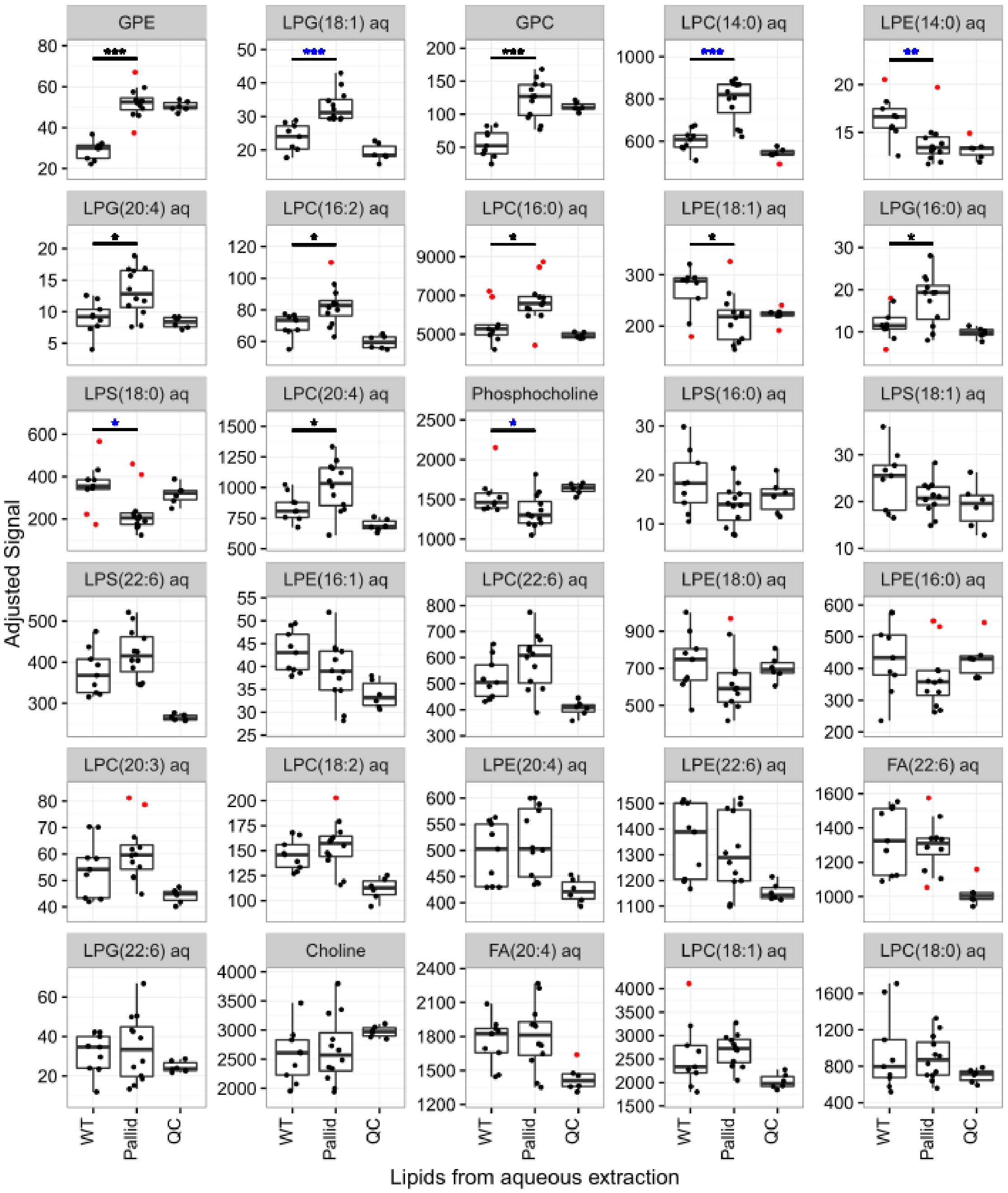

Figure S4) Boxplots for lysophospholipids and lipid derivatives from the aqueous extractions. QC: quality control. Signals are the QC-corrected, normalized (MFC method) areas of manually integrated peaks where the sn1 and sn2 signals were summed. Significance levels: *** $p < 0.001$, ** $p < 0.01$, * $p \leq 0.05$. Black stars, Student's Two Sample t-test; blue stars, Wilcoxon rank sum test.

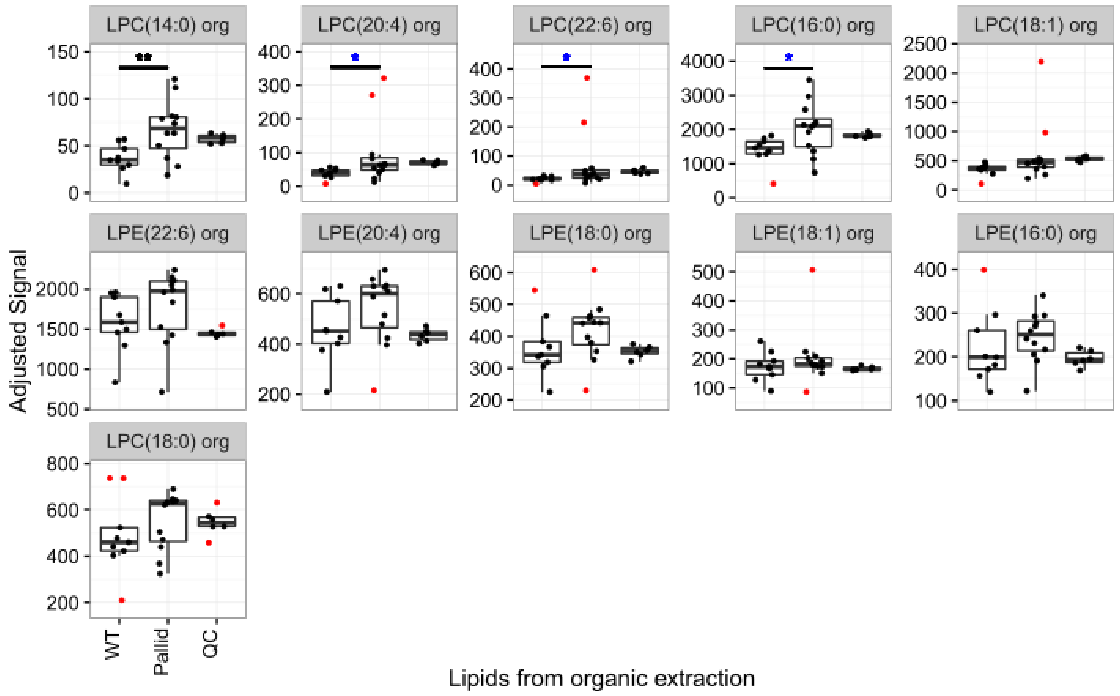

Figure S5) Boxplots for lysophospholipids from the organic extractions. QC: quality control. Signals are the QC-corrected, normalized (MFC method) areas of manually integrated peaks where the sn1 and sn2 signals were summed. Black stars, Student's Two Sample t-test; blue stars, Wilcoxon rank sum test.

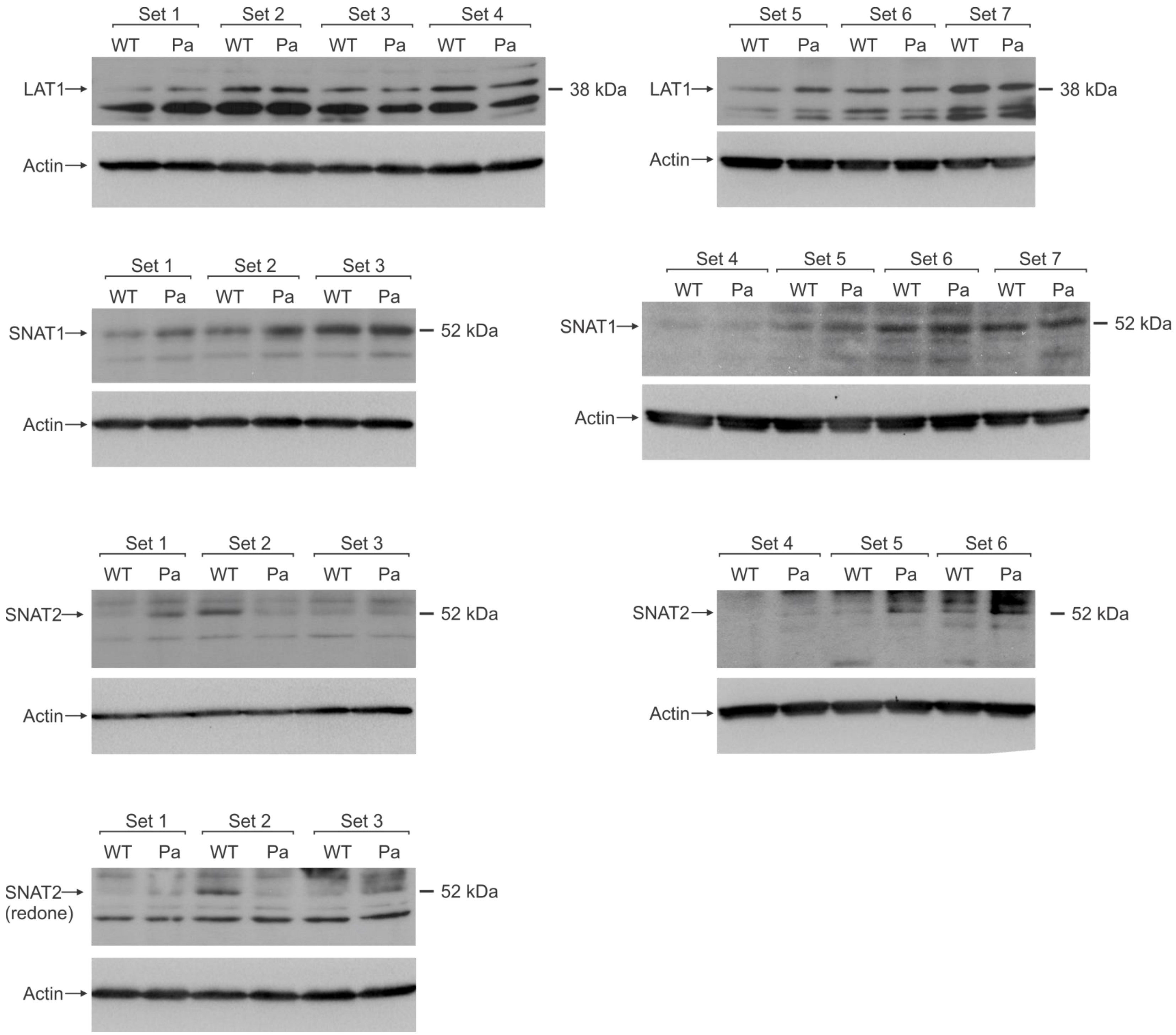

Figure S6) All western blots used for quantitation of relative protein levels of the amino acid transporters SNAT1, SNAT2 and LAT1. Arrows point at bands corresponding to the apparent molecular mass of each transporter (as per the technical information provided by the supplier of the commercial antibodies).

Actin bands for LAT1 set2,3,4 are the same as for SNAT2 (redone) Set1,2,3 because they were ran on the same membrane. Quantitative data for all bands is included in sheet 2 of supplemental Excel workbook.

Supplemental 2 – R-script

```r
#####################################################################################
#                                                                                   #
#  A heuristic function to clean up noise features from an LCMS data matrix.      #
#                                                                                   #
#  Based on the frequency of repeating m/z's (f)                                    #
#   within in a certain mz window (mzW)                                             #
#   over a defined RT window (rtW)                                                  #
#   below a certain signal strength (sgn).                                          #
#                                                                                   #
#  Takes a data matrix (obj) with samples in rows and features (mzrt-pairs)        #
#    in columns and with corresponding RT-values in 1st row and mz-values          #
#    in 2nd row. Moreover this data matrix  must be sorted (increasing)            #
#    firstly on mz-values and secondly on RT-values.                               #
#                                                                                   #
#                                                                                   #
#                                                                                   #
#####################################################################################



noise_reduction <- function(obj, mzW, rtW, f, sgn) {
  #gives column numbers from sorted data-matrix of repeated m/z's within a window to     create
intervals
  x   <- obj
  rmx <- rbind(c(1, rep(0, (ncol(x) - 1) ) ), c(rep(0, ncol(x) - 1), ncol(x) ) )
  for (i in 1:ncol(x) ) {
    for (j in i:ncol(x) ) {
      if ((x[2, j] - x[2, i ]) >= mzW )
      {
        rmx[2, i]<- j ; rmx[1, (i + 1) ] <- j  ; break
      } else {
        if (j == ncol(x) - 1 ) { rmx[2, i]<- j ; rmx[1, (i + 1) ] <- j
        } else {
          rmx <- rmx
        }
      }
    }
  }

  #creates matrix containing range (pos1/pos2), frequency and mean signal strength of IDs to be
removed
  rmx  <- rbind(rmx, apply(x,2, function(a) mean(a[3 : nrow(x) ],  na.rm = TRUE) ) )
  rmx1 <- rmx[ , rmx[1 , ] != rmx[2 , ] ]
  dRT  <- apply(rmx1, 2, function(a) (range(x[1 ,a[1] : (a[2] - 1) ] )[2] - range(x[1, a[  1]:(a
[2] - 1) ] )[1] ) )
  Smax <- apply(rmx1, 2, function(a) (max(rmx[3, a[1]:(a[2] - 1) ] ) ) )
  rmv  <- rbind(rmx1[-3, ],dRT,Smax) ; rownames(rmv) <- c("pos1", "pos2", "dRT", "Smax")
  rmv  <- as.matrix(rmv[  ,( (rmv["pos2", ] - rmv["pos1", ] - 1) >= f & rmv["dRT", ] > rtW & rmv
["Smax", ] < sgn) | rmv["Smax", ] == 0] ) #deletes empty columns



  #Remove features
  if (ncol(rmv) > 0) {
    rmv <- matrix(rmv[ ,(order( rmv["pos1", ], decreasing = T) ) ], nrow=4 )
    clx <- x
    for (i in 1:ncol(rmv)) {
      clx <- clx[ , -(rmv[1, i]:(rmv[2, i] - 1) )]
    }
  } else {clx <- x}
  return(clx)
}
```

Supplemental 3 – Additional Methods and Results

Materials & Methods

Chemicals

The HPLC grade solvents methanol (MeOH) and acetonitrile, water and chloroform were ordered from Sigma-Aldrich (Sigma Aldrich Química S.A., Madrid, Spain). The chemical standards of the test mix: acetaminophen, erythromycin, leucine-enkephaline (Leu-Enk), reserpine, sulfadimethoxine, sulfoguanidine, terfenadine, Val-Tyr-Val and verapamil, and metabolites: adenosine, adenosine monophosphate, arachidonic acid, choline, glutathione (oxidized, GSSG), glutathione (reduced, GSH), guanosine, guanosine monophosphate, inosine, L-arginine, L-aspartic acid, L-glutamic acid, L-glutamine, L-histidine, L-isoleucine, L-leucine, L-lysine, L-methionine, L-phenylalanine, L-proline, L-serine, L-tryptophan, L-tyrosine, methylthioadenosine, N-acetyl-aspartyl-glutamate, phosphocholine uridine, and uridine monophosphate were also ordered from Sigma-Aldrich. The chemical standard N-acetyl-aspartate as well as the mobile phase additive formic acid were obtained from Fluka (Sigma Aldrich Química S.A., Madrid, Spain). S-adenosyl-L-methionine was a kind gift from Abbott S.r.l. (Campoverde di Aprilia, Italy). The lipids 1-hexadecanoyl-sn-glycero-3-phospho-(1'-rac-glycerol) sodium salt (LPG(16:0)), 1-stearoyl-2-hydroxy-sn-glycero-3-phospho-L-serine sodium salt (LPS(18:0)), 1-tetradecanoyl-sn-glycero-3-phosphocholine (LPC(14:0)) and 1-octadecanoyl-sn-glycero-3-phosphocholine (LPC(18:0)) were obtained from Avanti Polar Lipids (Alabaster (AL), USA).

Antibodies for Western blots

The primary rabbit polyclonal anti-LAT1/SLC7A5 (PA5-50485; 1:300) was purchased from ThermoFisher, Waltham, MA; while, the mouse monoclonal antibodies were from EMD-Millipore Corporation, Billerica, MA, anti-SNAT1/SLC38A1 (clone N104/37; 1:100); Santa Cruz Biotech. Inc., Santa Cruz, CA, anti-SNAT2/SLC38A2 (G-8; 1:100) and Sigma, St. Louis, MO, anti-β-actin (1:10000). Horseradish peroxidase (HRP)-conjugated secondary antibodies were from Cell Signaling Technology, Danvers, MA.

Sample preparation for LC-MS analysis

Whole hippocampus preparations were transferred to of PRECELLYS® tubes containing zirconium oxide beads of 1.4 mm (Bertin Technologies, Montigny-le-Bretonneux, France). Next, 500 µL of ice cold 50% (v/v) methanol/water was added and the samples were homogenized in one cycle of 15 seconds at 4000 rpm in a PRECELLYS® 24 bead homogenizer and immediately chilled on ice. 450 µL of the resulting homogenate was transferred to 1.5 mL microcentrifuge tubes (Eppendorf, Madrid, Spain) and centrifuged for 10 minutes at 14,000 rpm and 4 °C. The supernatants were transferred to new tubes and evaporated to dryness in a speedvac for approximately 3 h. The resulting pellets were resuspended in 100 µL 50% (v/v) MeOH/water and centrifuged for 5 minutes at 14,000 rpm and 4 °C just before use. These samples are further referred to as the aqueous extractions.

The pellets obtained after homogenization and removal of the supernatant were further processed in 1 mL of a 3:1 (v/v) chloroform/MeOH mixture by vortexing for 30 minutes at 1400 rpm and 4 °C. The resulting suspension was centrifuged for 10 minutes at 14,000 rpm at 4 °C, and the supernatant evaporated to dryness and resuspended in 100 µL MeOH. The resuspensions were centrifuged for 5 minutes at 14,000 rpm and 4 °C just before use. These samples are further referred to as the organic extractions.

Quality control samples (QCs) for both aqueous or organic extractions were obtained by pooling 25 µL of each sample in their respective extraction groups yielding two QC samples of about 250 µL. Six control samples (three for each extraction method) were prepared by following the exact extraction protocol but in the absence of tissue. These extraction controls (ECs) were used to determine and eliminate signals that originated from plastics, solvents and other non-biological sources.

Liquid chromatography and mass spectrometry

Samples were analysed on a UPLC system (Acquity, Waters Inc., Manchester, UK) coupled to a time-of-flight mass spectrometer (ToF-MS, SYNAPT G2, Waters Inc). The MS was operated in both positive and negative electrospray ionization (ESI+ /-) modes with capillary voltages of respectively 500 V and 1 kV. The extraction cone voltage was 6 V and sampling cone voltage was 35 V. Source temperature was 120 °C while capillary temperature was 450 °C. Two scan functions were used: one for low collision energy (4 V) and one for high collision energy (25 V), both with scan times of 0.1 seconds. Samples were measured in full scan mode from 50 to 1200 Da. Drift in *m/z* values was corrected for by the lock mass of a Leu-Enk signal at *m/z* 556.2771. Both polarities were tuned on the Leu-Enk signal to a mass resolution of 20,000 FWHM which corresponds to an average mass error of less than 1 mDa.

All samples were separated on a 1 mm x 100 mm, 1.7 µm BEH C18 UPLC column (Waters Inc.), thermostated at 40 °C. Solvent A consisted of 99.9% water, 0.1% formic acid while solvent B consisted of 99.8 % acetonitrile, 0.1 % water and 0.1 % formic acid. The gradient was as follows: from 100 %A to 0 %A in 7 minutes in a linear gradient, constant at 0 %A for 3 minutes and back to 100 %A in 0.1 minutes and 2.4 minutes to re-equilibrate the column. The flow rate was 140 µL/min and the injection volume was 5 µL.

Injection sequences consisted of test mixes, extraction controls, initialization runs, randomized sample sequences and QCs. First the LC and MS performance was evaluated by injecting a test mix containing acetaminophen, sulfoguanidine, sulfadimethoxine, Val-Tyr-Val, verapamil, terfenadine, Leu-Enk, reserpine and erythromycin all in a concentration of 2 µM. Then the extraction control samples were injected. Next the column was equilibrated by injecting 6 to 8 times in succession from the QC vial. After equilibration the randomized sample sets were injected. Before the samples and after each 6 samples and at the end of each run a QC sample was injected in order to check the stability of the system. In total 6 QC samples per run were recorded. The aqueous and organic sample sets were first measured in ESI+ mode and then in ESI- mode.

Data analysis

Definitions. Each variable provided by LC-MS analysis is defined by a retention time (RT) on the chromatographic column and a mass/charge (*m/z*) ratio generated by the mass spectrophotometer. In order to differentiate between an identified *m/z*-RT pair (mzRT) and an mzRT without identification, different indicators were used. The most representative mzRT (normally the de(protonated) species or a salt adduct) for a metabolite was referred to simply as a metabolite. However, when the identity of an mzRT was (still) elusive it was referred to as a feature. Features can be (de)protonated species, (salt) adducts or fragments. A marker was defined as a metabolite or a feature that significantly differed between sample groups.

Feature extraction. Extracted ion chromatograms (XICs) were obtained from raw LC-MS data by MarkerLynx (ML) software (Waters Inc., Manchester, UK). All *m/*z values between 50 and 1200 Da and RT values between 0.40 (t_0) and 8.50 minutes were collected. XICs were obtained in a mass window of 10 mDa. Peaks in the XICs were determined with the *apex track* option with smoothing applied. Peaks were collected in a mass window of 200 mDa, a RT window of 0.1 minutes and with an intensity area bigger than 200. Isotopes and noise signals were removed (noise elimination level 6, black-box algorithm). It should be mentioned that while the threshold in the ML software is given as the integrated peak area the resulting feature tables that were used for further analysis contained the non-normalized peak heights. The average *m/z* values with their corresponding RTs and intensities (peak height) per sample were tabulated for four separate data sets *i.e.* ESI+/aqueous extraction (Pos/Aq), ESI+/organic extraction (Pos/Org), ESI-/aqueous extraction (Neg/Aq) and ESI-/organic extraction (Neg/Org).

Quality control samples (QCs) for both aqueous or organic extractions were obtained by pooling 25 µL of each sample in their respective extraction groups yielding two QC samples of about 250 µL. Six control samples (three for each extraction method) were prepared by following the exact extraction protocol but in the absence of tissue. These extraction controls (ECs) were used to determine and eliminate signals that originated from plastics, solvents and other non-biological sources.

Data cleaning. Non-endogenous features, introduced by the extraction procedure (*e.g.* plasticizers, solvent contaminates) were eliminated from the data matrices. This was done by eliminating all features that were exclusively present in the EC samples above a height intensity threshold of 300. Next, features that showed no signals in QC samples were purged. Subsequently, data sets were further cleaned for background noise signals by applying a heuristic filtering method based on mass window, signal repetition frequency and intensity. The R-code for this filter is included in the Supplementary section (Supplemental 2). As a final cleaning step, only the feature with signals in more than 50 % of the samples for both genotype (*i.e.* > 4 for WT and > 6 for pallid) were retained.

Data pre-treatment. Where necessary, feature signals were corrected for signal drift during analysis. This was done by calculating the relative change in intensity in QC samples for each feature for the individual data sets (*i.e.* Pos/Aq, Pos/Org, Neg/Aq and Neg/Org) where the first QCs of the data sets were set to 100%. By using robust linear regression (rlm function, MASS package, R) a linear equation was derived between injection number and relative change. If the p-value for the modelled linear change of a particular feature was below 0.01, the values were corrected, based on their injection number. Subsequently, sample profiles (*e.g.* detected features per LCMS-run) were normalized by the median fold change (MFC) method[1,2]. The target profile was chosen to be the one exhibiting the highest total sum in all data sets except Neg/Org

(P11 in Fig. 1). However it has been shown that the choice for the target is irrelevant for the performance of this method.

Biomarker selection and identification. Markers were selected by null hypothesis significance testing but prior to this, features were checked for normality by the Shapiro-Wilk test. Based upon the resulting P-value for a particular feature either a two sample t-test ($P_{Shapiro\text{-}Wilk} > 0.05$, two-tailed) or a Wilcoxon signed rank test ($P_{Shapiro\text{-}Wilk} \leq 0.05$, two-tailed) was used on this feature. Then, depending on the Bartlett test, either equal or unequal variances were used for mean difference testing ($P_{Bartlett} < 0.05$). A significance level of $\alpha = 0.01$ for differences in means was used as a selection criterion for a potential marker from automatically processed data.

Next, an attempt at identification was made for the selected markers. In order to do so, elemental compositions based on *m/z* and isotope distribution were matched with database hits from the Human Metabolome Database (HMDB, http://www.hmdb.ca), Kyoto Encyclopedia of Genes and Genomes (KEGG, http://www.genome.jp/kegg) and The Scripps Research Institute (METLIN, https://metlin.scripps.edu/index.php) metabolite databases. Moreover, MS/MS spectra in the METLIN database were compared with the low and high collision energy spectra of the samples. When identification was not possible, the feature was excluded for further analysis.

Supervised integration with QuanLynx (QL, Waters Inc.) software was performed by manually determining the baselines of XIC-peaks. Reintegration of markers was cross-performed between extraction phases of similar polarities. The areas were again normalized and drift-corrected and the corrected areas were used for subsequent analysis. Adducts and fragments were identified and removed from the final marker sets.

Because automatic peak integration was suboptimal and thus increasing the chance of omitting markers, KEGG pathway mapping was used to enrich the marker set. First, pathways were identified in which the reintegrated markers occurred. Then the *m/z* values of the (de)protonated species and/or sodium adducts of these nearest neighbors were used to look for peaks in their corresponding extracted ion chromatograms (XICs). When XIC peaks were detected they were integrated with QL. When the number of options for the metabolic identity of a marker was sufficiently narrowed down, chemical standards (when available) were used to determine its identity with higher confidence.

Software. For statistical analysis and data clean up the SIMCA P+ EZinfo module (MKS Data Analytics Solutions (formerly Umetrics), Umeå, Sweden) in Markerlynx (Waters Inc.), Microsoft Office Excel 2010 and custom R scripts (R 3.2.3, R Core Team, 2015) were used. The following R packages were used: ggplot2, cowplot, ggbeeswarm, dplyr, tidyr, readxl, car, psych and MASS. Graphs and figures were further enhanced with Inkscape 0.91 and GIMP 2.8.16.

Results

Metabolomics data analysis pipeline performance

Data pre-treatment

Data cleaning. After obtaining the four initial data sets, raw data needed to be cleaned of non-endogenous features and features due to background noise. It was important to assure that all features came from endogenous sources, because the total amount was later used for normalization. Thus all the features detected in the extraction controls (ECs) were deleted from the datasets. As a result, the number of features was reduced by 26% (Pos/Aq) to 69% (Pos/Org). Further unwanted features (mainly background ions) were deleted with a heuristic filter, thereby reducing the remaining number of features by another 5% to 20% (Table S1, Supplemental 1 - Fig. S1). The remaining features that did not contain any signals in the QC samples were deleted as well. Finally, features for which more than 50% of the values were missing per genotype-group were also deleted. After the cleaning process, between 67% (Neg/Aq) and 91% (Pos/Org) of the initial features had been purged (Table S1).

Data adjustment. Since individual analytes showed linear drifts in intensity along the analytical run, which could have been due to a plethora of causes (e.g., degradation, precipitation), data had to be corrected. Therefore, QC samples were used to detect and, if necessary, correct this signal drift. On average, about 2% of the features from the four cleaned-up data sets had to be corrected. These QC-corrected values were then used for normalization.

It is widely accepted that for metabolomics studies it is important to normalize the samples in order to minimize possible inter-sample differences in tissue amount, extraction efficiency and MS-source performance. We tried different methods for data normalization, namely normalization based on the sum of the peak heights of all features (sum method) and normalization based on the median fold change observed in each profile (MCF method). The latter method reduced the median of the CV values of the features per sample group more than the sum method and the CV values of the QC samples were less affected (Supplemental 1 Fig. S2).

After normalization, a rough quality check on the cleaned and adjusted data sets was performed with principal component analysis (PCA). Based on the resulting PCA scores, the spread in QCs for the Pos/Aq, Pos/Org and Neg/Aq sets was lower than that of the samples, indicating that the variation was mainly due to biological causes (Fig. 1). However, the spread in QC scores of the Neg/Org set matched that of the samples, indicating that not all the variability observed for this particular dataset was due to biological causes.

Marker selection.

Marker selection was performed in three stages. First, raw, auto-integrated data were used to obtain a preliminary set of differentially expressed features by means of univariate statistical analysis (Feature Markers, Table S1). Features from this set were assigned a preliminary identification based on exact mass, isotope distribution and fragmentation patterns. Selected features without identification, background noise signals, fragments and salt adducts were

discarded and only true metabolite signatures were retained (Metabolite Markers (auto), Table S1).

Next, the metabolite signals were manually reintegrated from raw LCMS data to obtain accurate peak areas and adjusted (QC-corrected, normalized) values were subjected to univariate analysis. Only one metabolite marker from the auto-integrated set was discarded, resulting in the detection of 14 unique metabolites (6 from Pos/Aq and 8 from Neg/Aq) in the aqueous extraction phase. Metabolite markers in this primary marker set could be roughly indexed in three groups, namely amino acids, nucleotides and lysophospholipids (Table 1 and Fig. 3). Significantly increased amino acid levels were found for aspartate, glutamate, glutamine, histidine and the neurotransmitter N-Acetylaspartylglutamic acid (NAAG), while lysine, phenylalanine and tryptophan were all significantly decreased. Among the nucleosides, adenosine and methylthioadenosine (MTA) were significantly upregulated in the pallid hippocampus (Fig. 3 and S3). Finally, glycerophosphocholine (GPC), glycerophosphoethanolamine (GPE) and the lysophosphatidylcholines (LPCs) LPC(14:0), LPC(16:0), LPC(20:4) were significantly upregulated (Fig. 3, S4 and S5).

The fourteen differentially expressed metabolites were then used to enrich the marker set by searching for associated metabolites. The search-by-association was performed by looking at close neighbours of the markers in the KEGG pathway database. Because the marker list contained amino acids, LC-MS data was also mined for all amino acid signals. Furthermore, a targeted search was performed for lysolipids associated with late endosomes, like lysophosphatidylglycerol (LPG) [3]. Enrichment led to the discovery of 14 extra metabolites that showed significant changes between genotypes. For both the organic and aqueous extraction phases LPCs and lysophosphatidylethanolamines (LPE) were detected after pathway enrichment (Fig. 2). However, only LPC exhibited upregulation in both phases, while LPEs were upregulated only in the aqueous phase. lysophosphatidylglycerol (LPG) and lysophosphatidylserine (LPS) were only detected and found to be differentially expressed in the aqueous extractions (Table 1). Diacylated lipid species could not be quantified because of the chromatographic method used.

Besides lysophospholipids, changes in levels of proline, N-acetylaspartate (NAA), uridine monophosphate (UMP) and phosphocholine were also found via pathway enrichment (Fig. 2, S3 and S4). A total of 28 metabolite markers (final column, Table S1) between extraction phases and polarities were found (Table 1 of which 14 were discovered by pathway enrichment. Moreover, another 29 metabolites discovered by pathway enrichment were not significantly changed by the pallid mutation. A graphical overview of all metabolites can be seen in Fig 2. Boxplots for all analysed metabolites based on adjusted signal intensities are included in the supplemental section (Supplemental 1, Fig. S3-S5). Detailed information about spectrometric properties and identifications of all included metabolites (markers and non-markers) can be found in Supplemental Data.

References.

1 Dieterle, F., Ross, A., Schlotterbeck, G. & Senn, H. Probabilistic quotient normalization as robust method to account for dilution of complex biological mixtures. Application in 1H NMR metabonomics. *Analytical chemistry* **78**, 4281-4290, doi:10.1021/ac051632c (2006).

2 Veselkov, K. A. *et al.* Optimized preprocessing of ultra-performance liquid chromatography/mass spectrometry urinary metabolic profiles for improved information recovery. *Analytical chemistry* **83**, 5864-5872, doi:10.1021/ac201065j (2011).

3 Meikle, P. J. *et al.* Effect of lysosomal storage on bis(monoacylglycero)phosphate. *Biochem J* **411**, 71-78, doi:10.1042/BJ20071043 (2008).

Supplemental 4 - Table

Table S1) Data metrics. Number of features left (and percentage discarded from Total) after cleaning, marker selection and pathway enrichment.

**Data set**[1]	auto-integrated raw data							manually reintegrated data		
	Total	*EC Purge*[2]	*Filter Purge*[3]	*QC Purge*[4]	*Minimal Observation Purge*[5]	*Feature Markers*[6]	*Metabolite Markers (auto)*[7]	*Metabolite Markers (manual)*[8]	*Unique Metabolite Markers*[9]	*Unique Metabolite Markers after Pathway Enrichment*[10]
**Pos/Aq**	6351	4729 (26 %)	4344 (32 %)	1269 (80 %)	757 (88 %)	44	12	11	6	10
**Neg/Aq**	1526	914 (40 %)	771 (49 %)	593 (61 %)	470 (69 %)	38	9	9	8	17
**Pos/Org**	4175	1278 (69 %)	977 (77 %)	509 (88 %)	340 (92 %)	15	0	0	0	1
**Neg/Org**	1300	658 (49 %)	404 (69 %)	269 (79 %)	203 (84 %)	6	0	0	0	0

1) Data sets are defined in materials and methods.
2) Purge features present in extraction controls (EC).
3) Purge features with heuristic filter.
4) Purge features that have no values in quality controls (QC).
5) Purge features with more than 50% missing data for both genotypes.
6) Features selected from adjusted, raw data with $p \leq 0.01$.
7) Preliminary identified metabolites from auto-integrated data
8) Metabolites selected from manually reintegrated and adjusted data with $p \leq 0.05$.
9) Unique metabolite markers between both electron spray ionisation modes (no markers present in organic extractions).
10) Unique metabolites between ESI modes and extraction phases.
